# Absence of Increase in Carotid Artery  Intima-Media Thickness in Infants of  Diabetic Mothers

**DOI:** 10.4274/jcrpe.v3i3.28

**Published:** 2011-09-09

**Authors:** Mehmet Emre Atabek, Havva Hasret Çağan, Beray Selver Eklioğlu, Bülent Oran

**Affiliations:** 1 Selçuk University School of Medicine, Department of Pediatric Endocrinology, Konya, Turkey; 2 Selçuk University, School of Medicine, Department of Pediatrics, Konya, Turkey; 3 Selçuk University, School of Medicine, Department of Pediatric Cardiology, Konya,; +90 332 223 63 50 berayselver@hotmail.com

**Keywords:** Intima-media thickness, left ventricular mass, macrosomic newborns

## Abstract

**Objective:** Infants of diabetic mothers (IDM) are considered as a risk group for atherosclerosis. Increased aortic intima-media thickness has been reported in IDM. The purpose of this study was to assess carotid artery intima-media thickness (CA-IMT), left ventricular mass index (LVMI)  and atherosclerotic risk factors in IDM.

**Methods:** Thirty IDM and 25 healthy controls were included in the study. Of these infants, 14 were appropriate-for-gestational age (AGA) and 16 were large-for-gestational age (LGA). CA-IMT and LVMI were obtained by M-mode  echocardiographic examination.  The relationship between parameters of atherosclerosis and echocardiographic measurements was assessed by Pearson’s correlation analysis.

**Results:** LVMI was higher in LGA IDM when compared to AGA IDM and controls. CA-IMT was not significantly different between the groups and was also not related to atherosclerotic risk factors. Serum lipid and insulin levels were higher in LGA IDM when compared with AGA IDM

and controls. There were no correlations between CA-IMT, LVMI and atherosclerotic risk factors.

**Conclusions:** In contrast to previous reports indicating an increase in CA-IMT in IDM, no differences were found between IDM and controls in this study. Our results indicate that  macrosomic IDM are prone to hypertrophic cardiomyopathy but not to atherosclerotic changes in the blood vessels.

**Conflict of interest:**None declared.

## INTRODUCTION

Diabetes mellitus during pregnancy affects fetal development adversely and causes metabolic disorders in neonates. The most important effect of diabetes on the fetus is macrosomia. The fetus will increase its insulin levels in response to the mother’s hyperglycemia  and this results in macrosomia (1). Alteration in the growth hormone/insulin-like growth  factor-1 axis is a risk factor for cardiovascular disease. Both hypersecretion and hyposecretion of growth hormone are known to increase the  risk for cardiovascular disease (2). Abnormalities in lipoprotein composition and concentration are also associated with macrosomia at birth and, elevated  lipoprotein levels which persist after birth may play a role in the development of atherosclerosis and diabetes in adult life (3). Clinical complications of atherosclerosis in  adulthood have a basis in childhood (4). Tracy et al (5) pointed to early  atheromatous changes in the  aorta. With modern technology, early vascular changes related to atherosclerosis can be imaged from peripheral vessels by sonography (6).

 In this study, we aimed to assess the risk of  subclinical  atherosclerosis by measuring carotid artery intima-media  thickness (CA-IMT) and left ventricular mass index (LVMI) in infants of diabetic mothers (IDM) by B-mode ultrasonography. Also, we investigated the relationship between CA-IMT and  cardiovascular risk factors.

## MATERIALS AND METHODS

This study was performed prospectively between December 2005 and December 2007 at Selcuk University, Meram School of Medicine, Pediatric Endocrinology Department. The study was approved by the local ethics committee. Signed informed consent was obtained from the parents. 

 Thirty  IDM and 25 healthy controls were included in the study. Fourteen of the IDM were appropriate-for-gestational age (AGA) and sixteen were large-for-gestational age (LGA). Gestational ages were determined by one or a combination of information based on mother’s last menstrual date, obstetrical ultrasound and Ballard score. 

Infants of mothers with a history of cardiovascular disease, high cholesterol level, hypertension, smoking, pre-eclampsia were excluded. Also, infants of mothers who had a history of taking drugs affecting lipid metabolism, such as steroids and ritodrine, were excluded. Infants with asphyxia, prematurity, respiratory distress, dysmorphic features, a stressful birth history and intrauterine infection were not included in the study. 

Neonates whose birth weights were above the 90th  percentile for gestational age and sex according to Lubchenco curves were accepted as macrosomic. IDM were divided into two groups as LGA and AGA.

 Weight was measured at birth by using digital scale and recorded in grams. Length and head circumference were measured using a standard board and tape and were recorded in centimeters. The ponderal index (PI) was calculated by the formula “weight (g) x 100/cm3.  A PI value over 2.85 was accepted as an index of LGA.

 Cord blood samples were taken from the study and  control groups. Cord blood samples received from Rh negative mother’s babies for blood group determination were used as the control group. Plasma triglyceride, total cholesterol,  high-density lipoprotein (HDL), low-density lipoprotein (LDL), very low-density lipoprotein (VLDL) and glucose levels were analyzed by colorimetric method (Beckman Coulter).  Plasma insulin and C-peptide levels were determined by chemiluminescence method (Immulite 2000, BioDPC, USA). Reference ranges accepted for insulin and C-peptide were 1.9-23 mIU/mL and 0.9-7.1 ng/dL, respectively. 

**Ultrasound Examination **

Ultrasound assessment of all infants was performed by the same clinician who was uninformed about the patient’s  laboratory values and risk factor levels. The subjects were  laid quietly for 10 minutes before the first scan in a dark,  temperature-controlled room. Scanning was performed in the supine position while the head was slightly extended and turned to the left. High-resolution B-mode ultrasonography of the right common carotid artery was performed by using a Philips Sonos 5500 Doppler  ultrasound with  3.5 MHz probe. Longitudinal images of the common carotid artery were obtained by combination of B-mode and color Doppler  ultrasound examinations. The IMT of the far wall of the  common carotid artery was measured with the electronic calipers of the machines, as described by Pignoli et al (7). 

 On a longitudinal two-dimensional ultrasound image of the carotid artery, the posterior  wall of the carotid artery  was shown as two echogenic lines. The external line was considered as the limit of medial adventitia and the inner  line - as the limit of the luminal intima. The distance between these two parallel lines shows the intimal medial thickness.  CA-IMT was calculated by taking the mean value of three measurements of maximum far wall thickness obtained from the common carotid artery, 10 mm below the carotid bulb.  

**Echocardiography **

Echocardiographic examination was conducted in all  infants in the postnatal 48-72 hours by the same clinician. Two-dimensional guided M-mode echocardiography (Philips Sonos 5500) was performed to determine LVM.  The ventricular  septal (IVSd) and posterior wall thicknesses at end-diastole (LVPWd), and the left ventricular end-diastolic (LVEDd) dimensions were determined according to the American Society of Echocardiography recommendations (8). LVM was calculated by using the Devereux formula (9):  0.8 * (1.04 * [(LVEDd+ IVSd + LVPWd) ** 3 - (LVEDd) ** 3)] + 0.6 We made a correction according to birth weight. LVMI was obtained by dividing LVM by birth weight. In our study, we  evaluated corrected LVM.

**Statistics**

All statistical analyses were performed using SPSS 13.0. Descriptive data were shown as mean±SD. Compliance with normal distribution was analyzed. Kruskal-Wallis variance analysis was performed for data which did not conform to a normal distribution. Bonferroni-corrected Mann-Whitney U test was applied for dual comparisons. For the relationship between CA-IMT and other parameters, Pearson’s 

correlation test was used.   A p-value lower than 0.05 was considered statistically significant.

## RESULTS

The clinical characteristics and laboratory findings of the IDM and control neonates are shown in Table 1. Mean birth weight, length, and head circumference were significantly high in LGA IDM  when compared to AGA IDM and controls. Among laboratory findings, only insulin levels were significantly high in the IDM groups.  LVMI also was higher in both AGA and LGA groups of IDM as compared to the controls. No differences in  CA-IMT were noted among the groups.   The correlations between anthropometric measurements, cardiovascular risk factors and CA-IMT are shown in Table 2, and the correlations between anthropometric measurements, cardiovascular risk factors and  LVMI are shown in Table 3. There were no significant correlations of anthropometric  measurements, lipid profiles, glucose, insulin and C-peptide  levels with either  LVMI or CA-IMT.

**Table 1:**
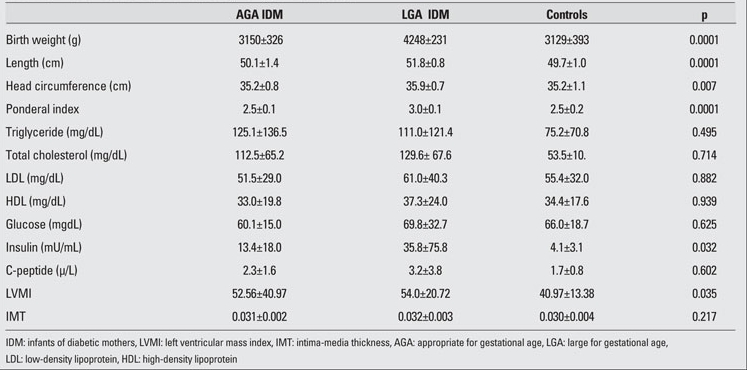
Table 1. Clinical characteristics and laboratory findings of the neonates

**Table 2:**
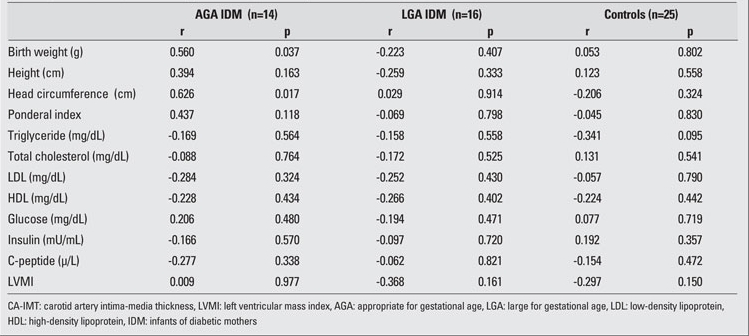
Table 2. Correlations between anthropometric measurements, cardiovascular risk factors and CA-IMT

**Table 3:**
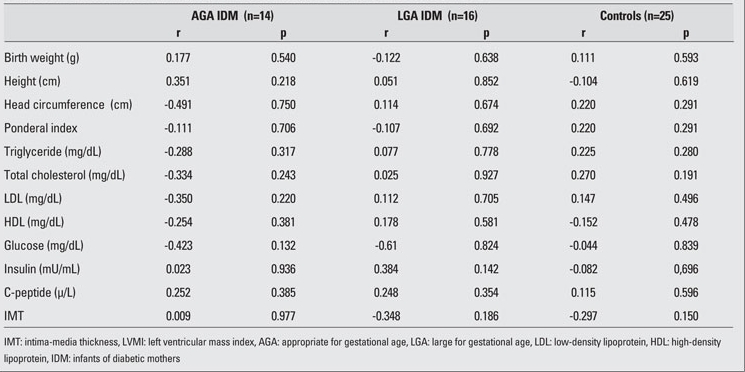
Table 3. Correlations between anthropometric measurements, cardiovascular risk factors and LVMI

## DISCUSSION

Fetal macrosomia and obesity are common complications occurring in IDM. Maternal hyperglycemia leads to fetal hyperglycemia which causes hyperinsulinism by stimulating the pancreatic islet cells in the fetus (10). The hyperinsulinemic status results in intrauterine growth of fat tissue, increased liver glycogen stores and  increased body mass. These changes are expressed as increased somatic growth, obesity and metabolic disturbances (11).

   Along with fetal hyperinsulinemia, macrosomia and  hypoglycemia, maternal hyperglycemia can also cause  asymmetric septal hypertrophy in the infant. Hyperinsulinemia induces increased synthesis of protein, glycogen and fat leads to hyperplasia and hypertrophy of the myocardium (12). Atherosclerosis is a chronic progressive process,  starting from the arterial wall and proceeding to the  obstruction of the lumen. Clinical signs and symptoms occur only in the advanced stages (13). Increased IMT of blood  vessels in peripheral arteries can be assessed with  B-mode ultrasonography in the early stages of atherosclerosis. Pignoli et al (7)  have demonstrated the compatibility of  ultrasonographic and histologic measurements of healthy and atherosclerotic carotid arteries . Akcakus et al (15) and Koklu et al 14,15) found significantly higher aortic IMT  values in macrosomic newborns and  considered this  finding  to be associated with  the atherosclerotic process (16). Today CA- IMT is accepted as an important parameter to be used in the assessment of patients who are at high risk for atherosclerosis. Atabek et al (17) reported increased  CA-IMT in diabetic children and adolescents. In these  studies, the carotid artery was chosen for assessment since it is easily visualized by ultrasonography. However,  atherosclerotic lesions usually start in the abdominal aorta. In our study, we found no differences in IMT values between IDM and control cases. The finding of non-increased IMT might be due to the fact that we used as parameter CA- IMT, which may not have been affected yet. Measuring carotid artery in neonates  without a linear probe and measuring a small area of the region constituted the difficulty of this study. The small number of subjects in our sample and the above-mentioned methodological problem were the main limitations of our study.

  Serum lipid, insulin, C-peptide and leptin levels were all reported to be elevated in IDM (18). We found no correlation between birth weight, height, head circumference, ponderal index and CA- IMT. 

Mehta et al (19) observed no differences between  macrosomic and non-macrosomic IDM by echocardiography, while Demiroren et al (20) reported that LVMI was the most important echocardiographic measurement for distinguishing macrosomic infants of diabetic and non-diabetic mothers. Akcakus et al (15) found an increased LVMI in macrosomic IDM. In our study, LVMI values were elevated in LGA IDM. Our results demonstrate that macrosomia and  hypertrophic cardiomyopathy in IDM are  associated findings. Increased levels of glucose, insulin, C-peptide and HbA1c in the cord blood of IDM  have been shown to be predictive  for the  clinical course of the infant as well as susceptibility to  atherosclerosis (21,22,23). In our study, in accordance with  previous reports, cord insulin levels were significantly high in IDM. On the other hand, cord C-peptide and glucose levels showed no differences compared to the values in the controls. These results indicate that hyperinsulinism is the most  effective parameter for macrosomia. Serum lipid levels were found high in IDM in our study, a finding reported in many studies (14,16,24,25).  A correlation between lipid profile and IMT has been shown in obese children in several studies (26,27). However, in this sample, we found no association between lipid profiles and CA- IMT. In conclusion, in the present study, increased LVMI values were found in IDM as compared to control cases, while no significant differences in CA-IMT were observed among the groups. From these results, we infer that macrosomic IDM may be prone to hypertrophic cardiomyopathy but not to  atherosclerotic changes in the blood vessels. 
